# Unraveling Chinese talk about emotion

**DOI:** 10.3389/fpsyg.2023.1157863

**Published:** 2023-08-16

**Authors:** Michelle Yik, Celia Z. Chen

**Affiliations:** Division of Social Science, Hong Kong University of Science and Technology, Hong Kong, Hong Kong SAR, China

**Keywords:** somatization, somatic symptom reporting, psychologization, emotion talk, Chinese culture

## Abstract

Research in cross-cultural psychiatry has asserted that Chinese people have a higher tendency to report somatic symptoms of their psychological distress than people with a European ethnic background. However, recent studies have reached inconsistent conclusions and most have confounded language use with culture in their study designs. Focusing on the varying degrees of orientation to Chinese culture, the present study examined the words freely listed by two Chinese groups of university students (mainland Chinese and Hong Kong Chinese) when describing their illness experience. Words were categorized into somatic, emotion, and somatic–emotion clusters. Overall, the Chinese participants were more willing to talk about their emotions than their somatic symptoms in an anonymous survey. The enculturated mainland Chinese participants—who reported greater Chinese cultural identity—used significantly more emotion words but fewer somatic–emotion words than the Hong Kong Chinese participants. No group differences were found in somatic words. In contrast to previous findings, the current study failed to find support for the relationship between orientation to Chinese culture and somatic symptom reporting when controlling for language use.

## Introduction

“Chinese are culturally trained to ‘listen’ within their body” (Ots, 1990, p. 26).

In the 1980s, the low prevalence of depression in Chinese communities caught the attention of researchers in cross-cultural psychiatry (Kleinman, 1986; see also Russell and Yik, 1996; Parker et al., 2001). Four decades later, an epidemiological study reported the lifetime prevalence of major depressive disorder as 3.4% in China (Huang et al., 2019), in comparison with 20.6% in the United States (Hasin et al., 2018) and 12.8% in Europe (Alonso et al., 2004). The data suggest that the community prevalence of depression among the Chinese population remains comparatively low. The rarity of depression among Chinese people is an area of active investigation (Yik, 2010), and somatization, the focus of the current study, has been offered as one possible explanation.

The low prevalence rates of depression may be partly due to the difficulties in defining and diagnosing depression in Chinese culture. In a seminal study, Kleinman (1982; see also Lee, 1999) found that neurasthenia, which emphasizes somatic symptoms such as fatigue, insomnia, and muscle pain, was a frequently used diagnosis in China (Ryder and Chentsova-Dutton, 2012). Kleinman found that Chinese patients diagnosed with neurasthenia could be re-diagnosed as having some level of depression using the Diagnostic and Statistical Manual of Mental Disorders III. Kleinman argued that neurasthenia is a potential somatic presentation of depression, which may account for the low rates of diagnosed depression in China during the 1980s (see also Ryder et al., 2008).

Although somatization appears to be a common phenomenon in Chinese communities and is not restricted to people with depression, some researchers have found that Chinese subjects are not alone in reporting somatic complaints. Parker et al. (2001) found that although a larger portion of Malaysian Chinese outpatients, compared with Euro-Australian outpatients, nominated a somatic symptom as their primary complaint (60% vs. 13%), neither group showed an exclusively emotion or somatic symptom profile. Ryder et al. (2008) found no differences between Chinese and Euro-Canadian outpatients in reporting somatic symptoms when privately responding to a questionnaire. When focusing on the individual items (rather than the scales composed of these items), Dere et al. (2013) found that their Chinese subjects reported high levels of affective symptoms, such as “suppressed emotions” and “depressed mood,” and spontaneously reported “depressed mood” at a level comparable to their Euro-Canadian counterparts.

Taken together, past studies have drawn inconsistent conclusions on the somatization phenomenon among Chinese people. Moreover, as most studies have compared one monolingual-monocultural group with another (e.g., Mandarin-speaking Chinese vs. English-speaking Canadians), language use has tended to be confounded with cultural background when testing somatic symptom reporting (see Kirmayer and Young, 1998). To assess the independent effect of culture on verbal expressions of illness experience, the present study recruited two Chinese samples that varied in their degrees of orientation to Chinese culture but used the same (Chinese) language.

### Conceptualizing somatization

“Emotions are thoughts somehow ‘felt’ in flushes, pulses, ‘movement’ of livers, minds, hearts, stomachs, skin” (Rosaldo, 1984, p. 143).

The notion of a close connection between emotions and somatic changes is not new in the literature. James (1884) was among the first batch of psychologists to propose that different emotions are associated with specific patterns of autonomic nervous system changes. Phrased differently, an emotional state can be shown via a pattern of physiological changes (Mauss and Robinson, 2009). The two-factor arousal theory also emphasizes the importance of physiology in the experience of emotion (Schachter and Singer, 1962).

Traditionally, somatization is a term tied to the psychodynamic notion that internal psychological conflict transforms into bodily distress (see Kirmayer, 1984; Lipowski, 1988). Some theorists have argued that somatization might be an outcome of the mind–body dualism inherent in a European ideology of medicine (Kirmayer and Young, 1998). From this dualist perspective, psychiatric disorders are seen as purely mental in nature and other medical conditions as physical (psychologization vs. somatization). Drawing on their random sample of 2,246 residents in a Canadian urban multicultural milieu, Kirmayer and Young (1998) concluded that somatization was ubiquitous in all cultural groups, although its prevalence and manifestation varied across cultures.

In recent research, somatization has been conceptualized in at least four different ways and its usage is often ambiguous. First, it has been regarded as the denial of psychological symptoms and substitution of somatic symptoms (Lipowski, 1988; Draguns, 1996; see also Parker et al., 2001). Second, it has been interpreted as “somatosensory amplification,” with the intense experience of bodily sensations giving rise to an emphasis on somatic symptoms (Zhou et al., 2011; see also Mak and Zane, 2004). Third, somatization has been defined as the presentation of somatic symptoms by people with psychiatric disorders (Simon et al., 1999; see also Tsai et al., 2004). Fourth, it has been portrayed as a form of help-seeking behavior through which people with somatic complaints are able to secure healthcare resources (Yen et al., 2000; Dere et al., 2013) or empathy.

### Somatization in Chinese culture

“Men can shed blood but not tears.”—Old Chinese saying.

The close connection between emotion and illness is still evident in everyday life in Chinese communities. An appointment with a Chinese doctor regarding a high blood pressure reading could result in a diagnosis of it being a symptom of stress, anxiety, or mood fluctuations. In this case, emotion is the cause of the high blood pressure, which is the somatic manifestation of stress or anxiety. Another classic example is *shenjing shuairuo* (neurasthenia), which is viewed as a somatic manifestation of depression associated with fatigue, insomnia, and muscle pain (Kleinman, 1982). The etiology of somatization can be traced back to ancient writings and medical practices in China (Kirmayer and Young, 1998; see also Yik, 2010).

The *Huangti Neiching* (The Yellow Emperor’s Classic of Internal Medicine; Veith, 1972), a compilation of ancient Chinese medical texts, applied the balance theory of emotion to the treatment of diseases. Somatic symptoms were diagnosed according to the affected organ from which the problematic emotion could be inferred. Extreme emotions were hypothesized to induce diseases (see Klineberg, 1938; Wu, 1982; Leung, 1998; Park and Hinton, 2002). In the *Neiching*, five emotions were mapped precisely onto five organs: anger was injurious to the liver, joy to the heart, worry to the lungs, fear to the kidneys, and contemplation to the spleen. If a patient had problems in these organs, the illness was thought to trigger excessive emotion, causing pain in other parts of the body (Saint Arnault, 2009) and thus worsening the patient’s overall health condition. In an investigation of the correspondence between emotions and bodily complaints in psychosomatic disorders at the Jiangsu Provincial Hospital, Ots (1990) found that patients tended to arrive with somatic complaints and that doctors avoided psychological diagnoses to prevent stigma. In fact, both parties preferred a somatic idiom based on the understanding that emotions lead to illness.

The close relationship between emotion and its somatic concomitants has been well documented in Chinese writing. Many emotion-related Chinese characters (ideographs) include a “heart” radical (see Russell and Yik, 1996). Tung (1994) found that many Chinese characters are rooted in the body, thereby accounting for the high prevalence of somatization among the Chinese. King (1989) investigated the ways in which Chinese people conceptualized emotions and concluded that metonymies—a part standing for the whole (e.g., smiling for happiness, crying for sadness)—play a significant role in the Chinese manifestation of the five emotion concepts. Relatedly, King found that *qi*, the energy flowing throughout all parts of the body, was highly related to emotion. In the *Neiching*, the relationship between emotion and qi was mapped carefully (e.g., anger makes qi rise; fright makes qi become chaotic). The emphasis on somatic symptoms, such as crying and pain in sadness, and the importance of qi in defining emotion are consistent with the phenomenon of somatization.

### Acculturation and somatization

The evidence presented thus far seems to suggest Chinese culture as a possible explanatory variable for the high prevalence of somatization. “Culture” can be broadly defined as a shared meaning system that includes values, rituals, beliefs, and language (Kroeber and Kluckhohn, 1952). Recent studies have focused on the relationship between acculturation and somatization. Acculturation is the process of psychological and cultural change that takes place as a consequence of interaction between cultural groups (Berry, 2005). For instance, a Chinese subject moving to the United States might become acculturated to the host culture by learning its values and rituals.

In their community sample of 1,747 Chinese immigrants in the United States, Mak and Zane (2004) failed to find support for a relationship between somatization and acculturation to U.S. culture. The immigrants’ somatization scores were instead correlated positively with age, distress severity, and psychosocial stress, and negatively with education levels and sources of social support (see Beirens and Fontaine, 2011). Similarly, in a community sample in Australia, Parker et al. (2005) found that Chinese subjects acculturated to Australian culture reported depressive episodes at a comparable level to that of a control group of matched non-Chinese. Yen et al. (2000) found that Chinese students endorsed lower levels of somatic depressive symptoms than Chinese subjects who identified with U.S. culture and U.S. subjects of European ethnic origins. Their Chinese subjects, when they sought counseling, somatized when they were in China but not when they were in the United States.

Tsai et al. (2004) evaluated how participants with varying degrees of acculturation to U.S. culture differed in their word use when describing their childhood experiences and conflicts in their romantic relationships. The results showed that the less acculturated (to U.S. culture) Chinese group used more somatic and social words than did the U.S. participants of European ethnic origins. The authors contended that the use of somatic words may have been related to the participants’ levels of acculturation to Chinese culture. The U.S. participants of European ethnic origins, however, did not use more emotion words than did their Chinese counterparts who were acculturated to U.S. culture.

Ryder et al. (2008) studied Chinese outpatients in China and Canadian outpatients of European ethnic origins in Canada using three modalities: spontaneous reports and clinical interviews completed by clinicians, and self-report questionnaires completed by the participants. The Chinese patients reported more somatic symptoms than the Canadian patients of European ethnic origins only when interviewed by (unfamiliar) clinicians (Dere et al., 2013; see also Zheng et al., 1986). In contrast, the psychologization effect (use of psychological symptoms) among the Canadians of European ethnic origins was evident in all three assessment methods. The authors concluded that European or North American psychologization, rather than Chinese somatization, was a culturally unique phenomenon (Kirmayer and Young, 1998).

In summary, the evidence attesting to the relationship between somatization and Chinese culture is mixed. Chinese participants appear to have a higher tendency than their counterparts of European ethnic origins to somatize when interviewed by clinicians, but they are less consistent in their somatization tendency when reporting symptoms in an anonymous survey. To further complicate the picture, most studies have adopted a monocultural-monolingual design (for an exception, see Tsai et al., 2004). As such, group differences could be explained by culture and/or language use.

### The present study

In the present study, we sought to disentangle the effects of culture and language use on somatization by recruiting mainland Chinese (MC) and Hong Kong Chinese (HKC) participants. Given that both MC and HKC are of Chinese origin, our method focuses on enculturation, which refers to the acquisition of the values, beliefs, behaviors, and social norms of one’s own culture (Kim, 2007). HKC, having undergone years of British influence, have been found to be more bicultural and less oriented to Chinese culture than the relatively monocultural MC (Ng and Lai, 2011). We hypothesized that the MC group would attain higher scores in orientation to Chinese culture than the HKC group (Hypothesis 1) and that the MC group would use more somatic words but fewer emotion words than the HKC group (Hypothesis 2).

## Method

### Participants

Two groups of undergraduate students studying at a university in Hong Kong were recruited *via* email and an announcement made on the SONA system. For the HKC (MC) group, we asked for volunteers who were born in Hong Kong (mainland China) and had lived there for most of their lives. The sample characteristics of the participants are presented in Table 1. On average, the HKC group (*N* = 82; 42 females; *M_age_* = 21) had lived in Hong Kong for 20 years (*SD* = 2.38) and all listed Cantonese as their first and strongest language; the MC group (*N* = 70; 33 females; *M_age_* = 20) had lived in mainland China for 17 years (*SD* = 3.10) and two thirds of them listed Mandarin as their first and strongest language.

**Table 1:** Sample demographics.

Variable	Hong Kong Chinese (*n* = 82)	Mainland Chinese (*n* = 70)
Age (in years)	*M* = 21 (*SD* = 1.73)	*M* = 20 (*SD* = 1.63)
Female	51%	47%
Place of birth		
Hong Kong	86.60%	7.10%
Mainland China	8.50%	91.40%
Other	4.90%	1.40%
Number of years living in		
Hong Kong	*M* = 20 years (*SD* = 2.38)	--
Mainland China	--	*M* = 17 years (*SD* = 3.10)
Mother tongue		
Cantonese	100.00%	28.60%
Mandarin	--	65.70%
Other	--	5.70%
Strongest language		
Cantonese	100.00%	27.10%
Mandarin	--	67.10%
Other	--	5.70%
Self-perceived health status^a^	*M* = 2.12 (*SD* = 0.53)	*M* = 2.17 (*SD* = 0.54)
Orientation to Chinese culture^b^	*M* = 3.77 (*SD* = 0.43)	*M* = 4.14 (*SD* = 0.42)
Cultural identity^b^	*M* = 3.43 (*SD* = 0.48)	*M* = 3.99 (*SD* = 0.50)
Language proficiency^b^	*M* = 4.43 (*SD* = 0.51)	*M* = 4.44; (*SD* = 0.49)

^a^Possible scores range from 0 to 3. ^b^Possible scores range from 1 to 5.

### Measures and procedure

A traditional Chinese version of the questionnaire and Cantonese instructions were used in the sessions for the HKC group; a simplified Chinese version and Mandarin instructions were used for the MC group.

#### A recent illness episode

Following the procedure used by Tsai et al. (2004), the participants were asked to recall a recent illness episode and were guided to relive the experience by indicating *when* they fell ill, *where* they were at that time, and *how long* the illness lasted. Recalling such an episode provided an opportunity for the participants to express both emotional distress and physical complaints. They were then instructed to list up to 10 words to describe their experience. On average, each participant listed seven words (*SD* = 2).

#### Orientation to Chinese culture

To measure the participants’ orientation to Chinese culture, 15 items were adopted from the abridged version (for Chinese) of the General Ethnicity Questionnaire (GEQC; Tsai et al., 2000). These items were translated into Chinese using the back-translation procedure. The GEQC was designed to test Chinese Americans’ acculturation to Chinese culture. We chose the 15 items that bore face validity for measuring enculturation to Chinese culture among our participants. Of the 15 items, 10 measured Chinese cultural identity (*α* = 0.80),^1^ with responses made on a 5-point Likert scale ranging from 1 (*strongly disagree*) to 5 (*strongly agree*). The remaining five items measured Chinese language proficiency (*α* = 0.59),^2^ with responses made on a 5-point Likert scale ranging from 1 (*at least*) to 5 (*at most*). Higher scores indicated greater orientation to Chinese culture.

#### Self-reported health status

Because Mak and Zane (2004) found that participants who somatized their psychosocial distress tended to have a poorer self-perceived health status, we measured and compared the health status between the two Chinese samples in the present study using the Chinese version of Goldberg’s (1978) General Health Questionnaire (GHQ-60; *α* = 0.94; see Chan, 1985). Our two groups did not differ in their perceived health status, *F*(1, 150) = 0.003, *MSE* = 463.223, *p* = 0.957. An additional analysis showed that there was no significant main effect or interaction effect involving gender.

## Results

### Enculturation to Chinese culture

An ANOVA was conducted to test whether there was a difference in the orientation to Chinese culture between the MC and HKC groups. The orientation to Chinese culture was stronger in the MC group (*M* = 4.14, *SD* = 0.42) than in the HKC group (*M* = 3.77, *SD* = 0.43), *F*(1, 150) = 29.345, *MSE* = 0.181, *p* < 0.001. There was a significant difference on the Chinese cultural identity subscale, *F*(1, 150) = 48.643, *MSE* = 0.239, *p* < 0.001, but not in the Chinese language proficiency subscale, *F*(1, 50) = 0.038, *MSE* = 0.252, *p* = 0.845. Additional analyses revealed no significant main effects or interactions involving gender. Consistent with Hypothesis 1, the MC participants had a stronger orientation to Chinese culture (*viz.* Chinese cultural identity) than did their HKC counterparts.

### Data reduction in word use

From our sample of 152 participants (82 HKC, 70 MC), we obtained 1,049 responses in total. To assess the word use, we used a bottom-up approach to code these spontaneous responses in Chinese (see Widen et al., 2011).

#### Step 1

Identical responses were grouped together. For example, all examples of the Chinese term *pijuan* (tired and sleepy) were grouped together and counted as one. However, terms such as *hen pijuan* (very tired and sleepy) were not grouped with pijuan (tired and sleepy) as the two terms are not identical. In this step, 510 unique responses were identified.

#### Step 2

Two coders categorized the 510 unique responses into groups. Two responses were put into the same group if they had identical core morphemes with the same lexical meaning or if they were synonyms in most contexts. For example, units such as pijuan (tired and sleepy) and hen pijuan (very tired and sleepy) were grouped together because they both have the core morphemes *pi* (tired) and *juan* (sleepy), representing the same lexical meaning, “tired and sleepy”; units such as pijuan (tired and sleepy) and *pibei* (tired and sleepy) were grouped together as they are synonyms that can be used interchangeably in most contexts. Disagreements in coding were resolved through discussion between the coders. In this step, 323 syntactic groups were identified.

#### Step 3

The 323 groups were further categorized into seven mutually exclusive clusters (i.e., each group could be assigned to one cluster only), which are presented in Table 2. Groups were clustered together only when they described the same type of state or process. For example, both the pijuan (tired and sleepy) group (e.g., pijuan, hen pijuan, and pibei), and the *toutong* (headache) group were placed in the “somatic” cluster because they described the participants’ physical states, whereas the *jiaolv* (anxious) group was placed in the “emotion” cluster (see Clore et al., 1987; Tsai et al., 2004). A “somatic–emotion” category was created to capture words that could be used to describe either somatic or emotion states depending on the context. For instance, *nanshou* (suffered) could mean “suffering physically” in a context such as *shenti nanshou* (feeling physically ill) and “suffering psychologically” in a context such as *xinli nanshou* (feeling miserable).

**Table 2:** Clusters of words for describing experience during a recent illness episode.

Cluster	Definition	% HKC	Examples for HKC group	% MC	Examples for MC group
1. Somatic	Describe sensory and perceptual processes as well as physical states and functions	22.56%	*lei* (tired), *pijuan* (tired and sleepy), *fali* (lacking strength), *toutong* (headache), *keshui* (sleepy)	17.78%	*pijuan* (tired and sleepy), *fali* (lacking strength), *lei* (tired), *teng* (aching), *mei weikou* (lacking appetite)
2. Emotion	Describe emotion states only	37.55%	*wuzhu* (helpless), *bu’an* (restless), *wunai* (speechless), *nanguo* (sad), *danyou* (worried)	49.49%	*wuzhu* (helpless), *danyou* (worried), *jiaoji* (tense), *haipa* (fearful), *gudu* (lonely)
3. Somatic–emotion	Describe either somatic or emotion states	19.31%	*xinku* (distressed), *tongku* (painfully distressed), *nanshou* (suffering), *bushi* (unwell), *mei jingshen* (lacking energy and spirit)	13.54%	*tongku* (painfully distressed), *nanshou* (suffering), *xinku* (distressed), *bushi* (unwell), *mei jingshen* (lacking energy and spirit)
4. Action tendency	Describe thinking process before taking actions	7.76%	*kewang binghao* (longing for recovery), *qidai* (expectant), *xiang gongzuo* (want to work), *xiang xiuxi* (want to rest), *buxiang shuohua* (do not want to talk)	7.47%	*hulue* (ignore), *qidai* (expectant), *kewang binghao* (longing for recovery), *xiang xiuxi* (want to rest), *shunqi ziran* (let it be)
5. Action	Describe actions taken	5.96%	*xiuxi* (rest), *chiyao* (take medicine), *shuijiao* (sleep), *heshui* (drink water), *gan project* (work)	3.43%	*deng* (wait), *qukan clinic* (go to clinic), *shuijiao* (sleep), *anwei* (comfort), *kan yisheng* (see the doctor)
6. General statements	Describe general states and external conditions	5.42%	*bei zhaogu* (be taken care of), *dai* (lethargic), *naodai kongbai* (blank mind), *bei guanxin* (be shown care and concern), *bingqing haozhuan* (illness condition improved)	5.66%	*bei zhaogu* (be taken care of), *jianqiang* (adamant), *xiguan* (get used to), *haozhuan* (condition improved), *nan’ao* (tough)
7. Others	Words that do not belong to any of the other categories	1.44%	*anggui* (expensive), *baizhou* (porridge), *taiwan* (Taiwan), *shenjing* (nerve), *shui* (water)	2.63%	*beizi* (quilt), *hongtangshui* (brown sugar water), *jiaren* (family), *jiaobu* (bandage), *jiaoluo* (a corner)

The total number of responses from the HKC group was 554 and from the MC group was 495.

All the data were coded into the seven word categories by the two coders independently. There was moderate agreement between the two coders’ categorizations, κ = 0.60 (95% CI [0.56, 0.63]), *p* < 0.001. We confirmed the final categorization after discussing the discrepancies. In tallying the responses, 80% were captured by the somatic, emotion, and somatic–emotion clusters in each sample. In both samples, the modal cluster was emotion. Paired *t*-tests revealed that both groups had a higher tendency to use emotion words than somatic words. Among the HKC participants, the percentage of emotion words (*M* = 39, *SD* = 26.87) was higher than the percentage of somatic words (*M* = 22, *SD* = 24.03), *t*(81) = 3.25, *p* < 0.01. Among the MC participants, the percentage of emotion words (*M* = 52, *SD* = 30.00) was higher than the proportion of somatic words (*M* = 17, *SD* = 20.90), *t*(81) = 6.11, *p* < 0.01. Contrary to past findings, our two Chinese groups did not emphasize more somatic terms than emotion terms in describing their illness experience.

### Variations in word use

In this section, we report on our test of whether there were differences in word use between the MC group and the HKC group when describing their illness (Hypothesis 2). We limited our analyses to the clusters of somatic words, emotion words, and somatic–emotion words. For each participant, we tallied the number of words categorized in each cluster and the total number of words listed. The proportion for each cluster was estimated and used as the dependent variable in subsequent analyses.

We first tested the relationship between the participants’ orientation to Chinese culture and word use when reporting their recent illness episodes. As shown in Table 3, the subscale of cultural identity was most strongly associated with word use: it was positively related to emotion words but negatively related to both somatic and somatic–emotion words. Chinese language proficiency was positively correlated with emotion words.

**Table 3:** Correlations between word use and other explanatory variables (*N* = 152).

Word Cluster	Orientation to Chinese culture	Cultural identity	Chinese language proficiency
Somatic	−0.18^*^	−0.19^*^	−0.08
Emotion	0.24^**^	0.23^**^	0.16^*^
Somatic–emotion	−0.16	−0.20^*^	0.01

^*^p < 0.05, two-tailed. ^**^p < 0.01, two-tailed.

**Figure 1:**
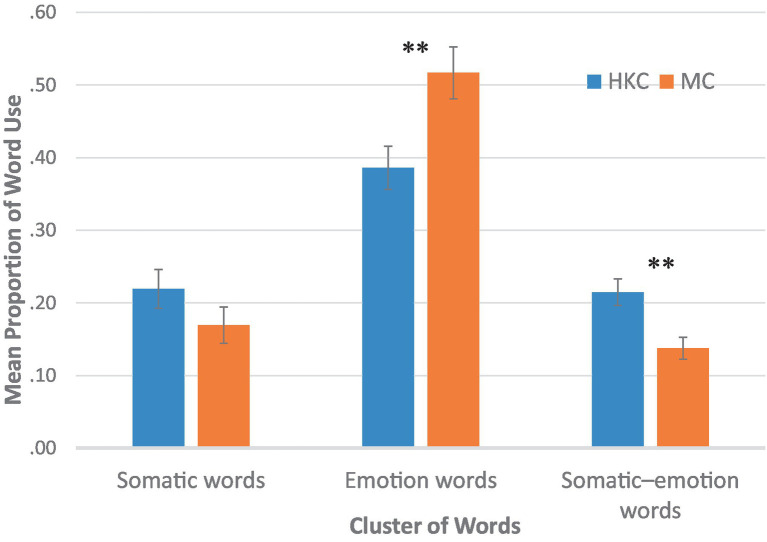
Use of somatic, emotion, and somatic–emotion words by group **p < 0.01, two-tailed.

We then conducted a series of ANOVAs on the word clusters. An ANOVA conducted on somatic words revealed no significant effect of Group, *F*(1, 150) = 1.836, *MSE* = 0.510, *p* = 0.177, although the HKC group had a higher tendency to use somatic words. A second ANOVA conducted on emotion words revealed that the main effect of Group, *F*(1, 150) = 8.036, *MSE* = 0.080, *p* = 0.005, was significant. The MC group used significantly more emotion words than the HKC group. A third ANOVA conducted on somatic–emotion words also revealed a significant main effect of Group, *F*(1, 150) = 10.044, *MSE* = 0.022, *p* = 0.002. The HKC group used significantly more somatic–emotion words than the MC group. Additional analyses were conducted to explore whether the group differences varied across gender and found that there were no significant main effects or interactions involving gender. The results are displayed in Figure 1. Contrary to Hypothesis 2, the (more enculturated) MC group did not use more somatic words or fewer emotion words than the (less enculturated) HKC group.

## Discussion

To better understand the conflicting findings on somatization and culture in the literature, we studied the words used by two Chinese groups with varying degrees of enculturation to Chinese culture (but with the same language) when discussing illness episodes. We found that both groups used emotion and somatic terms when describing their illness episodes and that both groups used more emotion terms than somatic terms. In contrast to our hypothesis and past findings, the (more enculturated) MC participants used more emotion terms and the (less enculturated) HKC used more somatic–emotion terms. No differences were found in the somatic terms.

### Somatization among Chinese people

We used a bottom-up approach in coding the open-ended responses. We created a cluster named “somatic–emotion” to categorize words that could be used to describe either somatic or emotional states (e.g., suffering, unwell) depending on the context. Past research has either lacked such a cluster or put these words under an “ambiguous” set (Ryder et al., 2008). A careful examination revealed that each somatic–emotion word could be interpreted as an integration of both somatic and emotion states. These words might provide an excellent window through which to unravel the somatization phenomenon. As this was the first time this cluster has been used in somatization studies, future research should be conducted to further investigate how useful these words may be for characterizing Chinese people’s verbal expression of emotions.

When reporting their experience of a recent illness, the (less enculturated) HKC participants used more somatic–emotion words. These words illustrate the traditional Chinese medical principles of focusing on symptom clusters that are each composed of somatic and emotion symptoms. We suspect that the HKC participants, although less oriented to Chinese culture, might still be prone to the influence of traditional Chinese medicine, which is widely practiced in both mainland China and Hong Kong (Lee, 1980; Hesketh and Zhu, 1997). Kleinman (1986) made a similar argument that somatization reflected the principle of traditional Chinese medicine. Chinese participants whose somatic states were conceptually closely associated with their emotion states tended to use more somatic words (or somatic–emotion words in the present study), even if they identified less with Chinese culture in general. In future studies, belief in traditional Chinese medicine can be examined as another explanatory variable to shed light on Chinese somatization.

### Somatization–psychologization dichotomy

In the 1980s, anthropologists and other social scientists asserted that somatization and culture were closely related. People who were more oriented to Chinese culture were said to report more somatic symptoms. During the subsequent four decades of extensive theoretical speculations and mostly monolingual-monocultural studies, somatization has been found to be related to many factors, such as acculturation, stress and anxiety, thinking style, and help-seeking behaviors.

In the present study, Chinese participants used twice as many emotion words as somatic words when describing an illness episode. The mainland Chinese participants—who highly identified with Chinese culture—preferred using emotion words to somatic words (49% vs. 18%). Echoing the conclusion of Ryder et al. (2008), researchers have perhaps spent too much time dichotomizing Chinese somatization and North American or European psychologization (see Kirmayer and Young, 1998). Although our Chinese subjects used both somatic and emotion terms, the more enculturated (to Chinese culture) subjects used more emotion terms than the less enculturated. Indeed, Dere et al. (2013) found that Canadian psychiatric patients of European ethnic origins endorsed high levels of atypical somatic symptoms, such as weight gain and appetite gain. Zhou et al. (2011) found that Chinese psychiatric patients had a higher tendency to emphasize somatic symptoms of depression, whereas Canadian patients of European ethnic origins had a higher tendency to emphasize somatic symptoms of anxiety (see also Ryder et al., 2008). Future work should be directed at finer-grained analyses using individual symptoms in lieu of scales (each composed of symptoms) and diverse types of emotion events. Relatedly, it would be prudent to move beyond the dichotomization of psychologization and somatization in characterizing cultures (see Kirmayer and Young, 1998).

### Enculturation to Chinese culture

To test the relationship between culture and somatization, we used two samples of Chinese participants who varied in their orientation to—and the extent to which they identified with—Chinese culture. We dichotomized the variable of cultural orientation using two criterion groups of students in a local university. In future studies, it would be worth actively recruiting non-student participants who vary along the dimension of cultural identity to further test the effect of cultural orientation on symptom presentation style (e.g., Tsai et al., 2004). Longitudinal studies are another alternative, as symptom reporting styles might vary as a function of a person’s length of stay in a place and/or acculturation to a certain culture.

To measure enculturation, we relied on the General Ethnicity Questionnaire. The items on this questionnaire do not define what Chinese cultural practices and customs are; rather, they tap the participants’ subjective perception of their adherence to Chinese culture, which is assumed to be consistent across the two Chinese groups. There is a possibility that “Chinese culture” was defined differently by the HKC and MC participants, and that these discrepancies could explain the present findings. To further the understanding of somatization in Chinese communities, future studies should be geared toward mapping the attributes of Chinese culture and testing how they contribute to somatization.

## Conclusion

Somatization is a multifaceted concept, the classical notion of which is a psychological problem being masked by somatic symptoms (Draguns, 1996). Researchers have also argued that somatization represents a help-seeking strategy to secure health care resources, to avoid stigma, or to reflect the unexplained somatic distress (see Kirmayer and Young, 1998; Parker et al., 2001). Our study used a free listing method in an anonymous survey to ensure that our participants would not put down any (somatic) symptoms for the purpose of seeking help or avoiding stigma. It appears that under this circumstance, Chinese people talk about emotions more than somatic symptoms. Psychological problems were not masked or overshadowed by somatic symptoms, and the group of more enculturated Chinese participants used more emotion terms than the group who were less enculturated.

## Data availability statement

The raw data supporting the conclusions of this article will be made available by the authors, without undue reservation.

## Ethics statement

The studies involving human participants were reviewed and approved by HKUST Human Research Ethics Committee. The participants provided their written informed consent to participate in this study.

## Author contributions

MY and CC designed the study together. CC coded the data and drafted part of the results section while MY wrote up the entire manuscript for publication. All authors contributed to the article and approved the submitted version.

## Funding

This study was funded by the Hong Kong Research Grants Council’s General Research Fund (Projects 16601818 and 16601921).

## Conflict of interest

The authors declare that the research was conducted in the absence of any commercial or financial relationships that could be construed as a potential conflict of interest.

## Publisher’s note

All claims expressed in this article are solely those of the authors and do not necessarily represent those of their affiliated organizations, or those of the publisher, the editors and the reviewers. Any product that may be evaluated in this article, or claim that may be made by its manufacturer, is not guaranteed or endorsed by the publisher.

## Footnotes

^1^The 10 items were (1) When I was growing up, I was exposed to Chinese culture. (2) Now, I am exposed to Chinese culture. (3) Compared to how much I negatively criticize other cultures, I criticize Chinese culture less. (4) I am embarrassed/ashamed of Chinese culture. (5) I am proud of Chinese culture. (6) Chinese culture has had a positive impact on my life. (7) I am familiar with Chinese cultural practices and customs. (8) I listen to Chinese music. (9) I celebrate Chinese holidays. (10) Overall, I am Chinese.

^2^The five items were (1) How much do you speak Chinese at home? (2) How much do you speak Chinese at school? (3) How much do you speak Chinese with friends? (4) How much do you view, read, or listen to Chinese on TV? (5) How much do you view, read, or listen to Chinese in literature?
